# Primary prevention of food allergy: beyond early introduction

**DOI:** 10.1186/s13223-024-00924-5

**Published:** 2024-12-19

**Authors:** Edmond S. Chan, Elissa M. Abrams, Douglas P Mack, Jennifer L.P. Protudjer, Wade Watson

**Affiliations:** 1 Division of Allergy, Department of Pediatrics, University of British Columbia, BC Children’s Hospital, Vancouver, BC Canada; 2 Department of Pediatrics, Section of Allergy and Clinical Immunology, University of Manitoba, Winnipeg, MB Canada; 3 Department of Pediatrics, McMaster University, Hamilton, Ontario Canada; 4 Halton Pediatric Allergy, Burlington, Ontario Canada; 5 Department of Pediatrics and Child Health, Rady Faculty of Health Sciences, Max Rady College of Medicine, University of Manitoba, Winnipeg, MB Canada; 6 Children’s Hospital Research Institute of Manitoba, Winnipeg, MB Canada; 7 Department of Food and Human Nutritional Sciences, Faculty of Agricultural and Food Sciences, University of Manitoba, Winnipeg, MB Canada; 8 George and Fay Yee Centre for Healthcare Innovation, Winnipeg, MB Canada; 9 Institute of Environmental Medicine, Karolinska Institutet, Stockholm, Sweden; 10 Department of Pediatrics, Division of Allergy, Dalhousie University, IWK Health Centre, Halifax, NS Canada

**Keywords:** Primary prevention, Food allergy, Early food introduction

## Abstract

Food allergy typically begins early in life and persists as a lifelong condition. Delayed introduction of allergenic foods followed by years of hesitancy to introduce these foods early may have contributed to the increase in food allergy prevalence in recent decades. Most infant feeding guidelines focus on the importance of early introduction of allergenic foods in infants at around age 4–6 months. However, regular, ongoing ingestion of allergenic foods is also critical for the primary prevention of food allergy. Similarly, intermittent exposure to cow’s milk formula (CMF) in early infancy increases the risk of cow’s milk allergy (CMA), while regular exposure (if it is introduced) prevents it. Families hesitant to introduce allergenic foods to their infant at home (despite education) should be offered introduction in a primary care clinic. Infants who have failed primary prevention should be referred to an allergist for consideration of early infant oral immunotherapy (OIT).

## Key-take home messages

• To reduce the risk of cow’s milk allergy (CMA), intermittent supplementation of breastfeeding with cow’s milk formula (CMF) in the first few months of life should be avoided. If introduced, ongoing regular supplementation (e.g., one bottle per day to supplement breastfeeding) is recommended to maintain tolerance.

• Infants should have allergenic foods (e.g., cooked [not raw] egg, peanut) introduced **at home**, at approximately 4–6 months of age (but not before 4 months) in high-risk infants and 6 months of age in low-risk infants.

• Once introduced and tolerated, it is essential that allergenic foods be eaten regularly (multiple times per month and at least once per week) in amounts representative of age-appropriate servings.

• A single exposure or occasional/intermittent exposures to allergenic foods should be avoided as this could be detrimental and result in food allergy.

• Families who are hesitant to introduce allergenic foods at home and in a primary care clinic should be referred to an allergist. Also, infants who have failed primary prevention should be referred to an allergist as soon as possible for consideration of early infant oral immunotherapy (OIT; see *Oral Immunotherapy* article in this supplement).

## Introduction

The prevalence of food allergy in Canada is approximately 6.1% [1]. Peanut, tree nut, sesame, fish, and shellfish allergies commonly persist beyond childhood, and severe reactions, including anaphylaxis, may occur [2–8]. A 4-year follow-up of peanut allergy in the population-based Australian HealthNuts cohort found that only 22% of 1-year-old children diagnosed with peanut allergy had resolution of their peanut allergy by age 4 years [5]. Low resolution rates have also been observed for tree nut (9–14%) [7], fish (0.6% per person-year in one study and 3.4-45% in another) and shellfish (0.8% per person-year) allergies [8, 9].

Although overall mortality due to food allergy is very rare, the fear of life-threatening anaphylaxis contributes significantly to the medical and psychosocial burden of this condition [10]. Evidence suggests that anxiety, and more specifically, food allergy anxiety, can be a significant burden for many children with food allergy and their families, which may contribute to psychological distress and functional impairment [11, 12]. Reduced health-related quality of life (HRQOL) has been observed among children and teens with food allergy, particularly older children and those with more severe manifestations [13]. Although the prevalence of food allergy in Canada does not appear to differ by income group or ethnicity [14], the limited evidence available supports that the burden related to food allergy is greater in some racial and ethnic communities and economically disadvantaged families compared to those who are White or economically-advantaged [15, 16]. The economic burden of food allergy is also substantial. One Canadian study found that the annual healthcare, out-of-pocket, and indirect (lost time and productivity) costs per individual with food allergy were $1267, $2136, and $7950, respectively [17]. It should be noted that this study collected data before the coronavirus disease (COVID-19) pandemic. Since the pandemic, food-related costs for families managing food allergy have increased [18].

Given the high burden associated with food allergy, primary prevention has become an important public health goal. This article will review current findings from observational studies, randomized controlled trials (RCTs) and meta-analyses that have led to recent guideline recommendations that go beyond early food introduction. According to these recommendations, the infant or child must regularly consume common allergens (at least weekly but ideally a few times per week) once introduced [19, 20]. Potential challenges in implementing guidelines are discussed, and key take-home messages for healthcare providers are provided. For details on infant oral immunotherapy (OIT) as a management option for failed primary prevention, please see the *Oral Immunotherapy* article in this supplement.

### Defining an infant “at risk” of developing food allergy

In 2017, the National Institute of Allergy and Infectious Diseases (NIAID)-sponsored guidelines defined an infant at “high risk” of peanut allergy as one with severe eczema and/or egg allergy, and an “at-risk” infant as one with mild or moderate eczema [10]. The most recent Canadian Paediatric Society (CPS)/Canadian Society of Allergy and Clinical Immunology (CSACI) practice guidelines define a “high-risk” infant as having either a personal history of atopy (e.g., eczema) or a first-degree relative (at least one parent or sibling) with an atopic condition (such as asthma, allergic rhinitis, food allergy, or eczema) [19]. The CPS/CSACI guidelines are also aimed at low-risk infants, and emphasize that food allergy can occur in infants with no specific risk factors, and that the mechanisms of sensitization are thought to be similar.

### Evidence supporting the early introduction of foods

The landmark Learning Early About Peanut (LEAP) study randomized 640 infants at high risk for peanut allergy to either early peanut ingestion (age 4–11 months) or avoidance (until age 5 years) and found an 86% reduction in peanut allergy with early and regular consumption of non-choking peanut-containing foods (2-gram servings three times per week) [21]. The study also found a preventative effect in both skin test-negative (13.7% vs. 1.9%; *p* < 0.001) and skin test-positive infants (35.3% vs. 10.6%; *p* = 0.004), supporting early peanut introduction as a means of both primary and secondary prevention.

The trials that have examined early egg introduction in high-risk infants have had conflicting results. The four RCTs that used pasteurized raw egg did not provide evidence of protection against egg allergy and/or reported more adverse events [22–25]. The only RCT to use cooked egg (Prevention of Egg Allergy with Tiny Amount Intake Trial [PETIT]) in infants with eczema found a significant reduction in egg allergy with earlier ingestion [26].

The Enquiring About Tolerance (EAT) study examined the early introduction of six allergenic foods (peanut, cow’s milk, sesame, fish, wheat, egg) in infants from the general population [27]. No significant difference in the rate of food allergy was found between the early-introduction (3 months) vs. standard-introduction (6 months) groups, likely because of the high rate of non-adherence to the dietary protocol. The Preventing Atopic Dermatitis and ALLergies in Children (PreventADALL) study randomized infants from the general population in Sweden and Norway to introduction of egg, milk, wheat and peanut by 3 to 6 months of age, early and regular emollient use, or both, and found that exposure to allergenic foods from 3 months of age significantly reduced food allergy at 36 months [28]. Early and regular application of emollients did not prevent either food allergy or atopic dermatitis. A recent systematic review and meta-analysis found “moderate certainty” evidence that introducing multiple allergenic foods from 2 to 12 months of age is associated with a reduced risk of any food allergy, but an increased risk of withdrawal from the intervention [29].

Regarding the optimal age of introduction in the first year of life, a secondary analysis of LEAP data showed that introduction after 6 months was associated with a higher likelihood of peanut allergy prevention (~ 95%) than introduction before 6 months (~ 85%) [30]. A recent study pooled data from EAT, LEAP and the observational Peanut Allergy Sensitization (PAS) study (which followed patients who were not eligible to participate in LEAP) to determine the optimal target populations and timing of peanut introduction to prevent peanut allergy in the general population [31]. The investigators found the greatest reductions in peanut allergy when the intervention was targeted to those with mild or no eczema. Also, different scenarios were generated based on the timing of peanut introduction, resulting in the following estimates of relative reductions in peanut allergy: 82% for all infants introduced at 4 months; 77% for infants with eczema introduced at 4 months and those without eczema introduced at 6 months; 58% for infants with eczema introduced at 4 months and those without eczema at 12 months; and 33% for all infants introduced at 12 months.

Data on the early introduction of other potentially allergenic foods, such as tree nuts, are sparse. Observational data from the Australian population-based longitudinal HealthNuts study found that no child who ate cashew by the age of 1 year developed cashew allergy, compared with 3.6% of those who had not consumed cashew by the age of 1 year [32]. An RCT focused on tree nut allergy prevention (TreEAT) is currently underway and will compare the efficacy and safety of a supervised multi-tree nut oral food challenge (OFC; almond, cashew, hazelnut, walnut) to standard care (home introduction of individual tree nuts) in infants 4–11 months of age with pre-existing peanut allergy (who are at high risk of developing tree nut allergy) [33].

### New insights into the prevention of immunoglobulin E-mediated cow’s milk allergy

Cow’s milk allergy (CMA) is the most common cause of fatal anaphylaxis among school-aged children [34]. Observational studies have reported an increased risk for developing CMA with delayed or irregular ingestion of cow’s milk early in life [35–37]. The Strategy for Prevention of milk Allergy by Daily ingestion of infant formula in Early infancy (SPADE) study found that ingesting a minimum of 10 mL of cow’s milk formula (CMF) at least once every day at age 1–2 months significantly reduced CMA at age 6 months compared with avoiding CMF supplementation [38]. The SPADE investigators also found that CMF supplementation did not compete with breastfeeding; approximately 70% of infants from both groups were still breastfeeding at 6 months of age. It should be noted that prior to age 1 month, both groups had frequent CMF exposure which suggests the importance of continued exposure once CMF is introduced.

The Cow’s Milk Early Exposure Trial (COMEET) is a recent interventional study that examined the association between early, continuous exposure to CMF (at least 1 bottle daily for a minimum of 2 months) and the development of immunoglobulin E (IgE)-mediated CMA in a large birth cohort from the general population [39]. The trial showed that, in the subset of breastfed infants given intermittent CMF (e.g., formula in the first few days of life followed by cessation of formula), the relative risk for developing CMA was 62.41 (3.27% CMA in the intermittently fed group vs. 0% in the daily CMF group; *p* = 0.01). Another recent analysis of COMEET found significantly higher rates of IgE-mediated food allergy during the first year of life in breastfed infants (2.9% in the exclusive breastfeeding group; 1.9% in the breastfeeding plus CMF group) compared to those who received only CMF (0%; *p* = 0.002) [40].

The latest CPS/CSACI position statement [19] advised that intermittent supplementation with intact CMF (e.g., a few bottles in the hospital followed by exclusive breastfeeding) should be avoided due to an increased risk of CMA, and when CMF has been introduced in an infant’s diet, it is important to ensure that regular ingestion of as little as 10 mL daily is maintained to prevent loss of tolerance. Interpreting the recommendation as ‘exactly’ 10 mL of CMF daily may be difficult to justify as it lacks practicality and raises concerns about formula wastage and cost. In light of these issues, a recent commentary by Canadian experts provided the following practical, real-world options: (1) exclusive breastfeeding; (2) extensively hydrolyzed formula (EHF) for intermittent supplementation; (3) full servings (i.e., 1 bottle per day) of intact CMF for ongoing regular ingestion once introduced [41]. For option 2, while EHF does not prevent allergic disease, intermittent use does not increase the risk of CMA. Partially hydrolyzed formula (PHF) is not recommended as intermittent exposure to PHF would expose the infant to enough cow’s milk protein to increase risk [41].

### Guidelines and issues related to their implementation

The most relevant food allergy primary prevention guidelines for Canadians are the CPS/CSACI recommendations [19], the North American Consensus Guidelines from the American Academy of Allergy, Asthma & Immunology (AAAAI), American College of Allergy, Asthma and Immunology (ACAAI) and CSACI [42], and the NIAID-sponsored guidelines [10] summarized in Table 1.

**Table 1:** Summary of guidelines for the primary prevention of food allergies [10, 19, 42]

	CPS/CSACI guidelines (2021) [19]	North American Consensus guidelines from the AAAI, ACAAI and CSACI (2020) [42]	NIAID guidelines (2017) [10]
**BF**	• BF for up to 2 years and beyond	• EBF recommended for all mothers • No association between EBF and the prevention of food allergy	• No specific recommendation provided • Guideline panel recognized that although early introduction of peanut may seem to depart from recommendations for EBF, evidence (LEAP) suggests that introduction of peanut does not affect the duration or frequency of breastfeeding
**Pregnant or BF mothers**	• Modifying the maternal diet to prevent food allergy not recommended (insufficient evidence)	• Maternal exclusion of common allergens not recommended • Use of any food supplement not supported	• No recommendation
**Introduction of food allergens**	• High-risk infants: introduce allergenic foods (e.g., cooked [not raw] egg, peanut) at about 6 months and not before 4 months of age • Low-risk infants: introduce allergenic foods at around 6 months	• All infants: introduce peanut and cooked hen’s egg starting around age 6 months but not before age 4 months • Do not delay the introduction of other allergenic foods (cow’s milk, soy, wheat, tree nuts, sesame, fish, shellfish) at around age 6 months but not before 4 months • New foods, including commonly allergenic foods, can be introduced on successive days, with no evidence of harm to this approach	• Highest risk infants (severe eczema and/or egg allergy): introduce age-appropriate peanut-containing food (see Table 2) as early as 4–6 months of age • Infants with mild-to-moderate eczema: introduce age-appropriate peanut-containing food around 6 months of age • Infants without eczema or any food allergy: freely introduce age-appropriate peanut-containing foods in the diet, together with other solid foods
**Continued intake**	• Once allergenic foods have been introduced, ensure regular, ongoing ingestion of age-appropriate serving sizes (i.e., a few times a week) to maintain tolerance	• No frequency, just add as regular part of diet	• Children who demonstrate tolerance to peanut, including those in the high-risk category, should eat peanut-containing foods regularly to maintain tolerance (i.e.,. 6–7 g of peanut protein [see Table 2] per week, divided into 3 or more feedings)
**Formula**	• When CMF has been introduced in an infant’s diet, make sure that regular ingestion (as little as 10 mL daily) is maintained to prevent cow’s milk allergy • For mothers who cannot or choose not to breastfeed, hydrolyzed formulas should not be recommended to prevent atopic conditions (e.g., eczema, asthma, allergic rhinitis) in either high- or low-risk infants	• Recommends against the use of any hydrolyzed formulas for prevention of food allergy or sensitization	• No recommendation
**Pre-emptive screening**	• Not recommended (risk of a severe reaction on the first exposure to an allergen is extremely low)	• Not required	• For high-risk infants, SPT or specific IgE blood tests recommended before introducing peanut

AAAI, American Academy of Allergy, Asthma & Immunology; ACAAI, American College of Allergy, Asthma and Immunology; BF, breastfeeding; CMA: cow’s milk allergy; CMF: cow’s milk formula; CPS, Canadian Pediatric Society; CSACI, Canadian Society of Allergy and Clinical Immunology; EBF, exclusive breastfeeding; IgE, immunoglobulin E; LEAP, Learning Early About Peanut Allergy study; NIAID, National Institute of Allergy and Infectious Diseases; SPT, skin prick testing

All three guidelines recommend the early introduction of allergenic foods (generally at age 4–6 months, depending on the guideline and infant risk level) and continued intake once introduced [10, 19, 42]. According to the CPS/CSACI guidelines, new foods, including commonly allergenic foods, can be introduced on successive days, with no evidence of harm to this approach [19]. Once common allergenic foods have been introduced, ongoing ingestion of age-appropriate serving sizes (i.e., a few times a week) is recommended to maintain tolerance. The NIAID guidelines advise that children who demonstrate tolerance to peanut consume 6–7 g of peanut protein (see Table 2 for peanut protein content of typical peanut-containing foods) per week, divided into 3 or more feedings [10].

**Table 2:** Typical peanut-containing foods, their peanut protein content, and feeding tips for infants [10]

	Peanut butter	Peanuts	Peanut flour or peanut butter powder	Bamba
**Amount containing approximately 2 g of peanut protein**	9–10 g *or* 2 teaspoons	8 g *or* ~ 10 whole peanuts (2½ teaspoons of grounded peanuts)	4 g *or* 2 teaspoons	17 g *or* 2/3 of a 28-g (1-oz) bag *or* 21 sticks
**Typical serving size**	Spread on a slice of bread or toast (16 g)	2½ teaspoons of ground peanuts (8 g)	No typical serving size	1 bag (28 g)
**Peanut protein per typical serving**	3.4 g	2.1 g	No typical serving size	3.2 g
**Feeding tips**	• For a smooth texture, mix with warm water (then let cool) or breast milk or infant formula • For older children, mix with pureed or mashed fruit or vegetables or any suitable family foods, such as yogurt or mashed potatoes	• Use blender to create a powder or paste • 2–2½ teaspoons of ground peanuts can be added to a portion of yogurt or pureed fruit or savory meal	• Mix with yogurt or applesauce	• For a smooth texture, mix with warm water (then let cool) or breast milk or infant formula and mash well • Pureed or mashed fruit or vegetables can be added • Older children can be offered sticks of Bamba

Bamba (Osem, Israel) is named because it was the product used in the LEAP trial and therefore has known peanut protein content and proven efficacy and safety. Other peanut puff products with similar peanut protein content can be substituted for Bamba

Teaspoons and tablespoons are US measures (5 and 15 mL for a level teaspoon or tablespoon, respectively)

Adapted from: Togias et al. 2017[10]

All guidelines support continued breastfeeding by mothers during the introduction of allergenic foods. The CPS/CSACI and North American Consensus guidelines do not recommend modifying the maternal diet (by avoiding or ingesting particular allergenic foods during pregnancy and while breastfeeding) to prevent food allergy given insufficient evidence to support such a recommendation [19, 42]. Both guidelines also state that there is insufficient evidence to recommend any supplement, such as vitamin D, omega 3, or pre- or probiotics, to prevent food allergies in infants. Although the North American Consensus guidelines recommend feeding infants a diverse diet to potentially prevent food allergy [42], the CPS/CSACI guidelines state that its role in preventing specific food allergies requires more research [19].

A key distinction between the three guidelines is their recommendations for pre-emptive food allergy screening. The CPS/CSACI guidelines argue against screening (i.e., skin or specific IgE testing prior to allergenic food introduction is “not recommended”) and the North American Consensus guidelines state that screening is “not required” [19, 42]. In contrast, the NIAID guidelines “strongly” advise allergy testing prior to peanut introduction in the highest-risk infants who have severe eczema, egg allergy, or both [10].

The CPS/CSACI approach to not screen even high-risk infants is based on pre-emptive screening for food allergy being poor utilization of limited resources due to its limited predictive value [43]. The high rates of clinically irrelevant positive results and long wait lists for infant OFCs in Canada to exclude false positives not only makes pre-emptive screening impractical, but also puts infants at risk of food allergy as they may miss the window of opportunity for primary prevention (i.e., by delaying the early introduction of allergenic foods). Poor cost effectiveness and the risk of ‘screening creep’ in lower risk infants are further impediments to pre-emptive screening for food allergy [44].

**Fig. 1:**
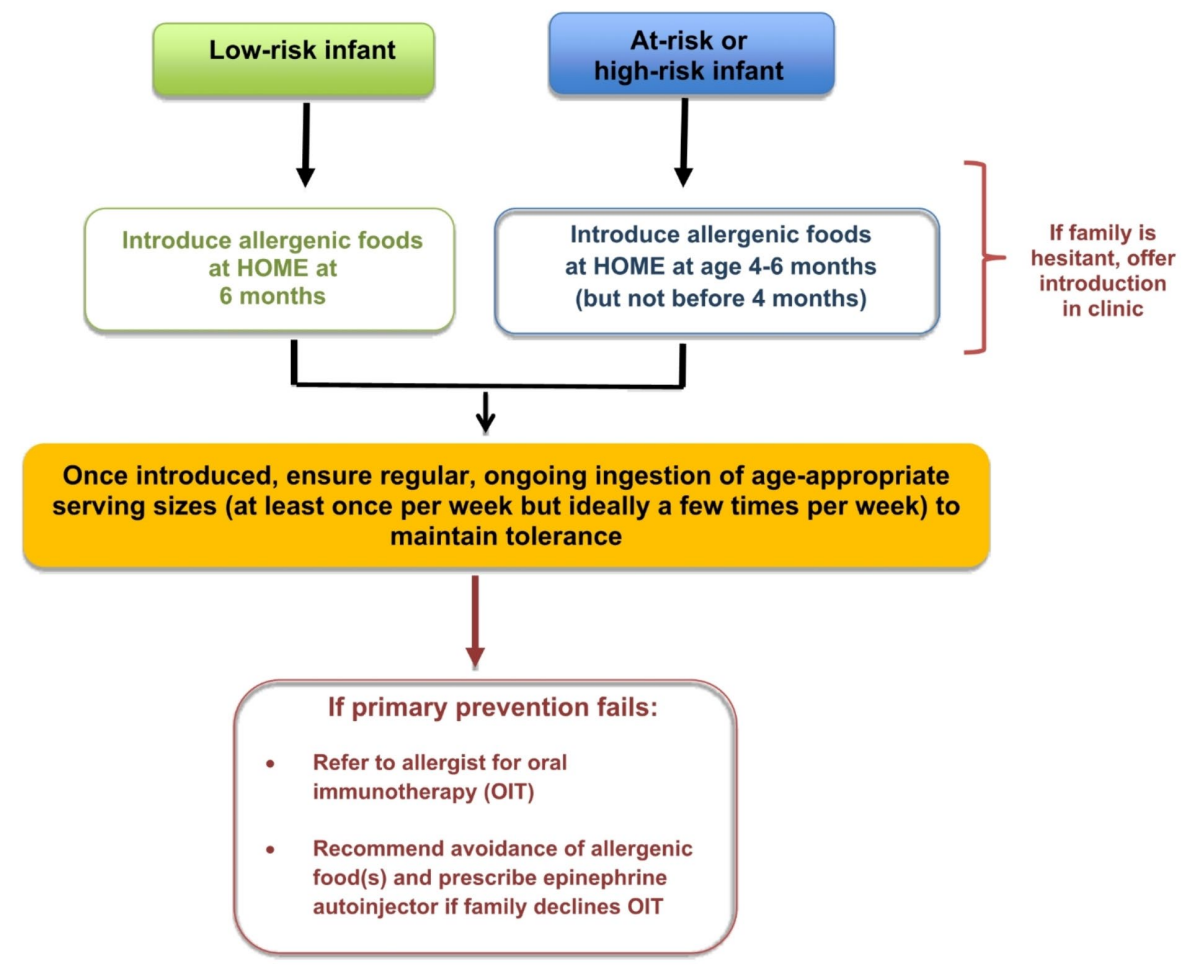
Simplified algorithm for the primary prevention of food allergy

The most cost-effective, practical and reliable way to introduce allergenic foods is to do so at home (see Fig. 1), which was especially brought to light during the COVID-19 pandemic. Evidence published during this time highlighted that home introduction is safe, even in high-risk infants, since the risk of a severe reaction upon first ingestion is extremely low [45, 46]. Families who are hesitant to introduce allergenic foods at home, despite proper education about the benefits of home introduction, should be offered introduction in a primary care clinic. If there is still hesitancy, then the family should be referred to an allergist (Table 3). For hesitant families, a novel approach (again brought to light during the COVID-19 pandemic) is virtually supported infant home introduction, which has been shown to be a practical and safe alternative to avoid delays in early introduction [47–49]. Infants who have failed primary prevention should be referred to an allergist as soon as possible for consideration of early infant OIT (see *Oral Immunotherapy* article in this supplement for more details on OIT).

**Table 3:** When to refer to an allergist

1) **Families hesitant to introduce commonly allergenic foods to an infant at home and in a primary care clinic for the purpose of primary prevention, despite proper education about the benefits of home introduction.** Example: At-risk infant whose family is hesitant to introduce non-choking peanut despite proper education
2) **Families of infants who already have a food allergy (suspected or confirmed)**,** who are hesitant to introduce other allergenic foods for the purpose of primary prevention.** *Example*: Infant with peanut allergy whose family is hesitant to introduce tree nuts or sesame in non-choking forms
3) **Initiation of infant OIT for failed primary prevention ***(see Oral Immunotherapy article in this supplement)*

OIT, oral immunotherapy

### The importance of regular ingestion once allergenic foods are introduced

For young infants (< 12 months of age), a fundamental component of the protocols of clinical trials such as LEAP and EAT was the regular ingestion (i.e., several times per week) of allergenic foods once introduced (see Table 4) [21, 27]. The COMEET trial (discussed earlier) provides clear evidence of what occurs when there is “interrupted” early introduction with ingestion of the allergen only intermittently. In this trial, the risk for developing CMA was significantly higher in the subset of breastfed infants given intermittent CMF (i.e., formula in the first few days of life followed by cessation of formula) compared to the group receiving daily CMF [40]. Once children are older (i.e., *≥* 23 months of age), findings suggest that a minimum of monthly ingestion of allergenic foods may be sufficient to maintain tolerance [50].

**Table 4:** Feeding protocols in the early introduction groups in the LEAP and EAT trials [21, 27]

Trial	Feeding protocol for allergenic foods
**LEAP** [21]	• 6 g of peanut protein per week, distributed in three or more meals per week
**EAT** [27]	• 2 g of each allergenic food protein twice each week (4 g of allergen protein per food per week) • Full weekly recommended amount for the allergenic foods consisted of: – Two small 40- to 60-g portions of cow’s milk yogurt – 3 rounded teaspoons of peanut butter – 1 small hard-boiled egg (< 53 g) – 3 rounded teaspoons of sesame paste – 25 g of whitefish – 2 wheat-based cereal biscuits (e.g., Weetabix)

From a public health perspective, there is now evidence confirming that early introduction of allergenic foods is not sufficient to reduce food allergy prevalence. The prevalence of peanut allergy (~ 3%) in infants in Australia has not changed when comparing 2007-11 with 2018-19 timeframes, despite a substantial increase in the proportion of infants (28–89%) fed peanut early since Australian infant feeding guidelines were updated in 2016 to recommend introducing peanut before age 12 months in all infants [51]. Similar findings were observed in a recent Swedish study [52]. An analysis of data from the population-based EarlyNuts study of 12-month-old infants in Australia found that while most families were introducing peanut in infancy, only ~ 30% of infants were eating peanut two or more times per week [53]. A large proportion were eating peanut less than once per week and some had even eaten peanut only once. Therefore, a lack of regular ingestion may be a key reason for the lack of change in food allergy prevalence despite early introduction.

**Fig. 2:**
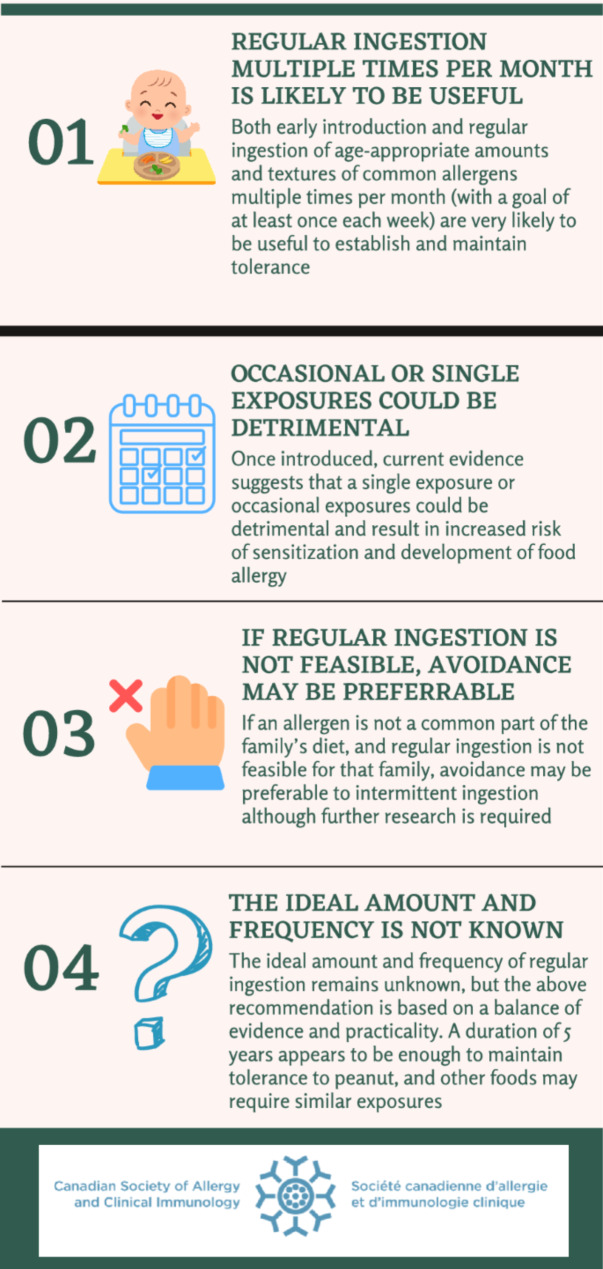
CSACI recommendations and considerations for the frequency of ingestion of allergenic foods to prevent food allergy [20]. Reproduced from Abrams 2023 [20]. See Creative Commons license at: https://creativecommons.org/licenses/by/4.0/ No changes have been made to this figure

Given the above-mentioned evidence, the CSACI has recently published a statement focused on the importance of ongoing regular ingestion of allergenic foods to prevent food allergy [20]. The CSACI recommends both early introduction and, once introduced, regular ingestion of age-appropriate amounts and textures of all common allergens multiple times per month (with a goal of at least once each week based on expert opinion) to establish and maintain tolerance (see Fig. 2). A duration of 5 years of ongoing regular ingestion appears to be sufficient to maintain tolerance to peanut, and other foods may require similar exposures. The CSACI advises against single or occasional exposures once allergenic foods are introduced, and recommends that if regular ingestion is not feasible, avoidance may be preferable to intermittent ingestion (e.g., some families do not consume shellfish regularly).

### Early introduction may be inadvertently promoting an increase in food protein-induced enterocolitis

Food protein–induced enterocolitis syndrome (FPIES) is a non-immunoglobulin E-mediated-food hypersensitivity that usually manifests in infancy and is characterized by repetitive emesis on ingestion of the culprit food (please see *Non-IgE-Mediated Food Allergy* article in this supplement). Although many foods are known to cause FPIES (most commonly cow’s milk, soy and grains [particularly rice and oats]), peanut- and tree nut-triggered FPIES has been emerging since the implementation of early food introduction guidelines [54–56]. A dramatic increase in FPIES provoked by hen’s egg has also been observed in Japan since guideline updates [57]. More research is needed to determine what makes certain infants more prone to FPIES than others, and whether concurrent primary prevention of both FPIES and IgE-mediated food allergy is possible. However, clinicians should avoid inadvertently increasing the risk of IgE-mediated food allergy by raising alarm bells about FPIES. Early solid food introduction should continue unabated given the relative abundance of data demonstrating that delayed introduction increases the risk of IgE-mediated food allergy [58].

## Conclusions

The increase in food allergy prevalence in recent decades is a major public health problem and may, in part, be due to years of delayed introduction of allergenic foods followed by hesitancy to introduce these foods early. Persistently high prevalence may be due to lack of regular ingestion once introduced. Recent findings from observational studies, RCTs, and meta-analyses now demonstrate that both early introduction and regular ingestion of commonly allergenic foods are imperative for the primary prevention of food allergy. Current Canadian guidelines recommend introducing allergenic foods (e.g., cooked [not raw] egg, peanut) at 4–6 months in high-risk infants and at around 6 months in low-risk infants [19]. Once introduced, ongoing ingestion (multiple times per month and at least once per week) is recommended to maintain tolerance [19, 20].

## Data Availability

Data sharing not applicable to this article as no datasets were generated or analyzed during the development of this review.

## Declarations

### About this supplement

This article has been published as part of Allergy, Asthma & Clinical Immunology, Volume 20 Supplement 03, 2024: Practical Guide for Allergy and Immunology in Canada 2024. The full contents of the supplement are available at https://aacijournal.biomedcentral.com/articles/supplements/volume-20-supplement-3.

## Abbreviations

AAP: American Academy of Pediatrics
AAAAI: American Academy of Allergy, Asthma & Immunology
ACAAI: American College of Allergy, Asthma and Immunology
BF: Breastfeeding
CI: Confidence interval
CMA: Cow’s milk allergy
CMF: Cow’s milk formula
COMEET: Cow’s Milk Early Exposure Trial
COVID: Coronavirus disease
CPS: Canadian Paediatric Society
CSACI: Canadian Society of Allergy and Clinical Immunology
EAT: Enquiring about Tolerance Study
EBF: Exclusive breastfeeding
EHF: Extensively hydrolyzed formula
HRQOL: Health-related quality of life
IgE: Immunoglobulin E
LEAP: Learning Early About Peanut study
NIAID: National Institute of Allergy and Infectious Diseases
OFC: Oral food challenge
OIT: Oral immunotherapy
PAS: Peanut Allergy Sensitization study
PETIT: Prevention of Egg Allergy with Tiny Amount Intake trial
PHF: Partially hydrolyzed formula
PreventADALL: Preventing Atopic Dermatitis and ALLergies in Children study
RCTs: Randomized controlled trials
SPT: Skin prick testing
SPADE: Strategy for Prevention of milk Allergy by Daily ingestion of infant formula in Early infancy study
